# Laser slice thinning of GaN-on-GaN high electron mobility transistors

**DOI:** 10.1038/s41598-022-10610-4

**Published:** 2022-05-05

**Authors:** Atsushi Tanaka, Ryuji Sugiura, Daisuke Kawaguchi, Yotaro Wani, Hirotaka Watanabe, Hadi Sena, Yuto Ando, Yoshio Honda, Yasunori Igasaki, Akio Wakejima, Yuji Ando, Hiroshi Amano

**Affiliations:** 1 Center for Integrated Research of Future Electronics (CIRFE), Institute of Materials and Systems for Sustainability (IMaSS), Nagoya University, Aichi, 464-8601 Japan; 2 National Institute for Materials Science, Tsukuba, 987-6543 Japan; 3 Research & Development Department, Electron Tube Division, Hamamatsu Photonics K. K., Shizuoka, 438-0193 Japan; 4 Department of Electrical and Mechanical Engineering, Nagoya Institute of Technology, Aichi, 466-8555 Japan

**Keywords:** Electrical and electronic engineering, Materials for devices, Techniques and instrumentation

## Abstract

As a newly developed technique to slice GaN substrates, which are currently very expensive, with less loss, we previously reported a laser slicing technique in this journal. In the previous report, from the perspective of GaN substrate processing, we could only show that the GaN substrate could be sliced by a laser and that the sliced GaN substrate could be reused. In this study, we newly investigated the applicability of this method as a device fabrication process. We demonstrated the thinning of GaN-on-GaN high-electron-mobility transistors (HEMTs) using a laser slicing technique. Even when the HEMTs were thinned by laser slicing to a thickness of 50 μm after completing the fabrication process, no significant fracture was observed in these devices, and no adverse effects of laser-induced damage were observed on electrical characteristics. This means that the laser slicing process can be applied even after device fabrication. It can also be used as a completely new semiconductor process for fabricating thin devices with thicknesses on the order of 10 μm, while significantly reducing the consumption of GaN substrates.

## Introduction

**Figure 1:**
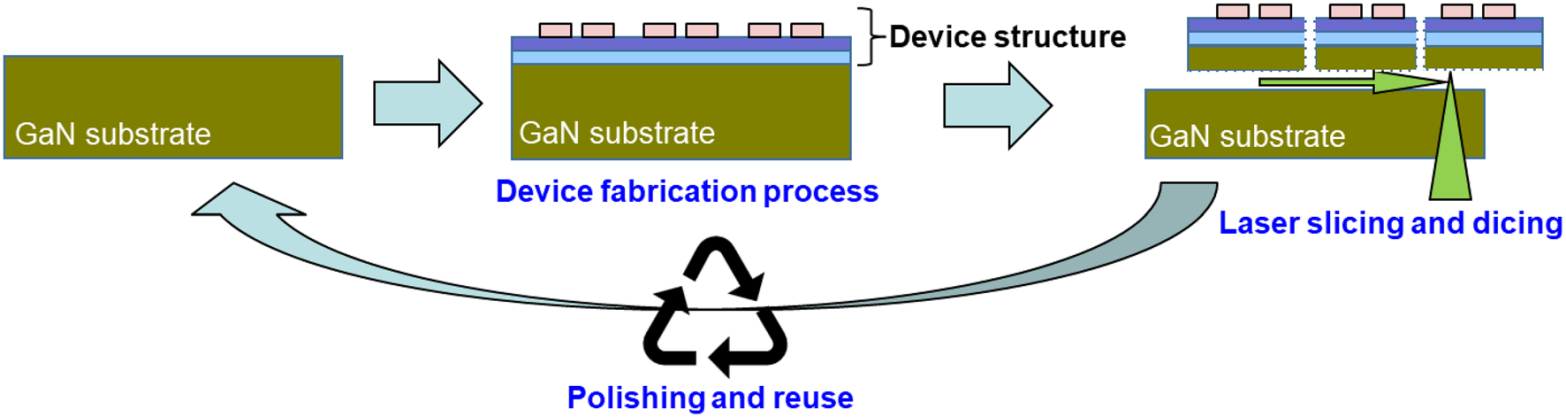
Schematic of device fabrication process with reusable substrates.

Gallium nitride (GaN) is a widebandgap semiconductor material and has especially many applications, among which light-emitting diodes (LED) and high-electron-mobility transistors (HEMTs) are already in practical use. GaN is also known to be a suitable material for power devices owing to its high critical electric field strength and high electron saturation velocity. Since heteroepitaxial GaN is available, it can be grown on Si, SiC, Al_2_O_3_, and other materials; however, the quality of crystals grown on GaN substrates is the highest, with the fewest crystal defects. It is expected that the use of high-quality crystals will lead to the higher yield and reliability of devices fabricated on GaN layers; therefore, the research and development of GaN devices on GaN substrates is also being actively pursued especially in the fields of next-generation power devices^1–19^ and high-power amplification of radio frequencies for the fifth-generation and beyond wireless communication^20–32^. However, owing to the high price of GaN substrates, GaN-based devices fabricated on GaN substrates have not yet been commercialized in a wide range of fields. To address this problem, we have developed a laser-based method of slicing GaN substrates^33,34^. This method not only suppresses kerf loss during the slicing of GaN substrates from GaN bulk crystals, but also minimizes the consumption of expensive GaN substrates if it can be used in processes such as that shown in Fig. 1. Since we have already confirmed that the laser-sliced substrate side can be reused after polishing, as reported in reference^33^, if the device can operate properly after this slicing, the amount of the GaN substrate consumed will only be the thickness of the sliced device layer and that removed by polishing for reuse. For example, as similarly indicated in reference^33^, if we remove a portion with a thickness of 50 µm as a device layer by laser slicing and remove another potion with a thickness of 50 µm by polishing to reuse the GaN substrate, we can obtain one device layer per 100-µm-thick GaN substrate, whereas previously, one device layer could only be obtained per 400-µm-thick GaN substrate. In addition, the thinner the device, the better the heat dissipation, making it desirable to reduce the thickness of the substrate under the device. The process shown in Fig. 1 is also convenient for obtaining thin devices. This laser slicing process reported here is similar to the Smart Cut™ technology^35–39^, if we look for something similar among conventional methods. Compared with the conventional Smart Cut™ technology, it is difficult to use this new laser slicing method to cut out a very thin GaN layer of submicron thickness because GaN is decomposed during laserslicing^33^. However, in our laser slicing method, the slicing plane can be formed by a laser, not by ion implantation. This allows processing at any position in the substrate. This means that we can peel off the device layer by irradiating laser from the backside surface after fabricating the device. Since laser slicing can be performed after device fabrication, it is possible for substrates to pass through the fabrication process as a single crystal. With this laser slicing, there is no need to develop methods of bonding to another substrate or to consider the chemical and thermal effects on the bonding interface during device fabrication.

In this study, we investigated whether the process shown in Fig. 1 is possible, i.e., whether the device can be sliced after the fabrication process, and, if so, whether the device will work properly after slicing. In this study, we focused on HEMTs as the devices to be thinned by laser slicing.

## Experiments

An Al_0.2_Ga_0.8_ N/GaN (20/900 nm) heterostructure was grown by metal organic vapor phase epitaxy (MOVPE) on a commercially available semi-insulating (SI) GaN substrate. Ohmic contacts were formed by evaporating Mo/Al/Mo/Au, followed by annealing at 850 °C in N_2_ ambient. As a field-plate dielectric, SiN was deposited by plasma-enhanced chemical vapor deposition. To electrically isolate each device, B^+^ ions were implanted. Various HEMTs were fabricated with different structural parameters; typically, the gate-to-source spacing, gate length, gate-to-drain spacing, and gate width were 2, 2, 5, and 100 µm, respectively. As electrode pads of the source, drain, and gate, Ni/Au with a size of 90 µm × 90 µm square was deposited. After measuring the electrical characteristics of on-wafer HEMT devices, dicing lines at intervals of 5 mm on the 50-µm-thick part of the device side were formed by the Stealth Dicing™ process^40^, and a slice plane was formed at a depth of 50 µm from the device side with a laser slicing technique. In both laser processes, the laser was irradiated from the back side surface of the sample, as shown in Fig. 1. The laser used for slicing in this study was a sub-nanosecond 532 nm green laser with a peak power density of 2.5 × 10^11^ W/cm^2^ and a beam diameter of 1 µm. To maintain the beam diameter even at a depth of 350 µm in GaN, this laser was equipped with a spatial light modulator (LCOS-SLM, Hamamatsu Photonics) to compensate for spherical aberration. The details of laser slicing conditions and procedure are described in reference^33^. Then, the electrical characteristics of thin-chip HEMT devices are measured again to evaluate the effects of laser slicing on the characteristics.

## Results and discussion

**Figure 2:**
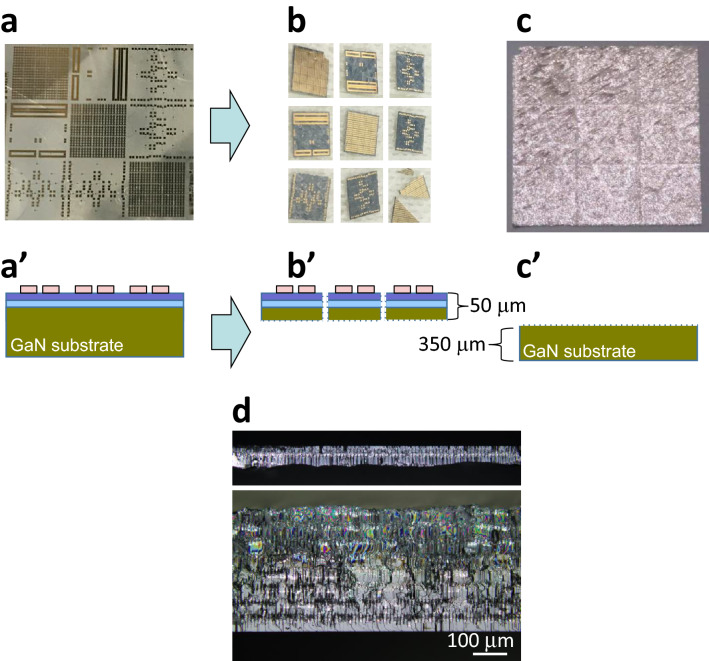
Photographs of sample before and after laser process. (**a**) As-fabricated on-wafer HEMTs. (**b**) 5-mm-square HEMT chip of 50 µm thickness. (**c**) 15-cm-square substrate of 350 µm thickness. a’, b’, c’ Schematics of cross-sectional structures of a, b, and c. (**d**) Cross-sectional photographs of sliced samples. Device-side sample (one of those in b, upper) and substrate-side sample (c, lower).

Figure 2 shows photographs of a sample before and after laser slicing. Figure 2a shows HEMT devices immediately after their fabrication on the SI-GaN substrate. Figure 2b shows nine sample chips with a thickness of 50 µm and a size of 5 mm square obtained by the Stealth Dicing™ process and laser slicing. Figure 2c shows the remaining 350-µm-thick, 1.5-cm-square SI-GaN substrate. The transparent area in Fig. 2b appears dark grey, and the surface of the GaN substrate in Fig. 2c appears silver, because Ga precipitated owing to the decomposition of GaN during laser slicing. In Fig. 2c, we can see grid like stripes at intervals of 5 mm, which are attached to the precipitated Ga and can be removed by appropriate cleaning. As shown in reference^33^, this part of the substrate can be reused as a substrate for epitaxial growth after polishing, indicating that the device fabrication process shown in Fig. 1 is possible.

**Figure 3:**
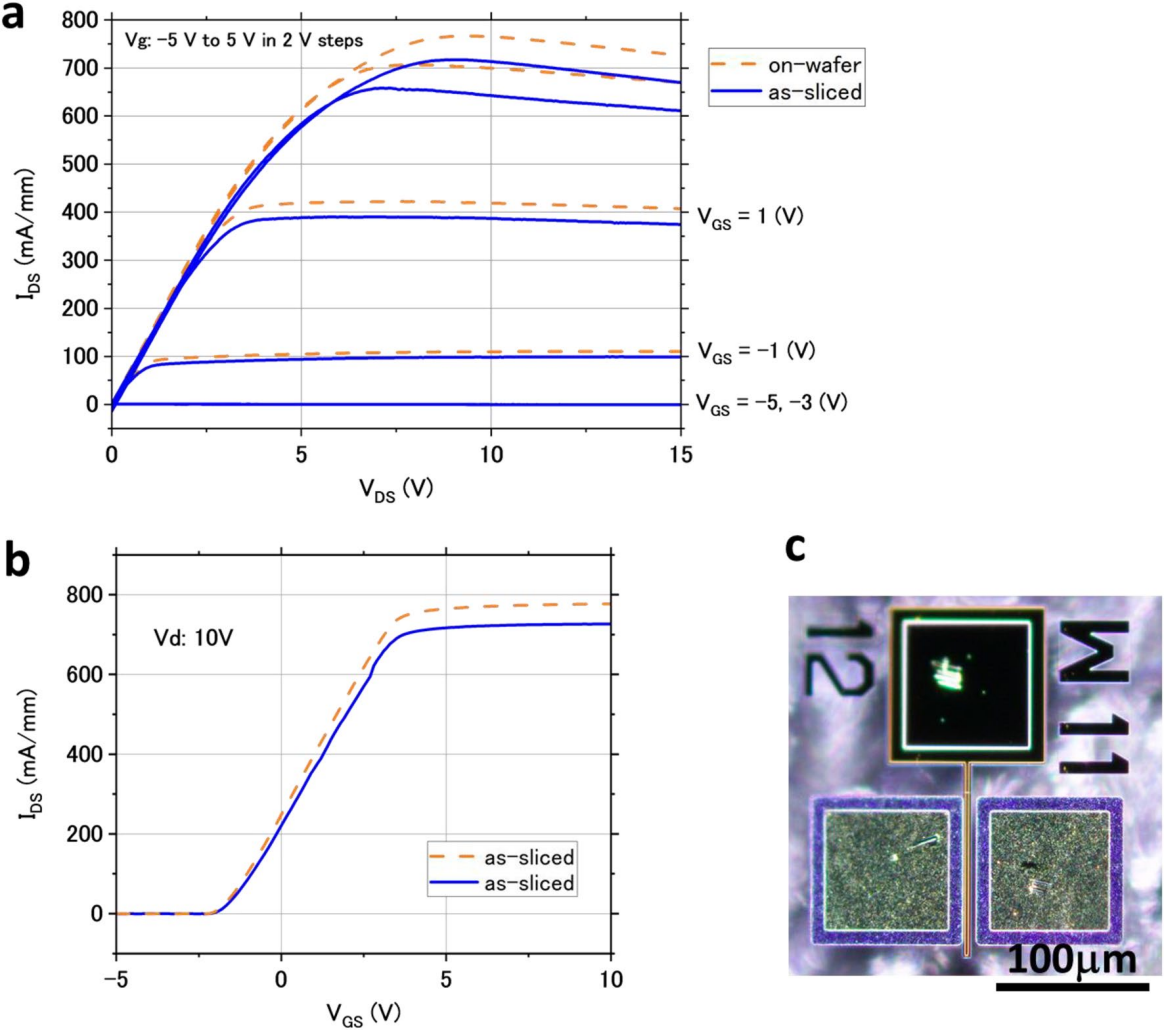
(**a**) I_DS_–V_DS_ and (**b**) I_DS_–V_GS_ curves of HEMT before and after laser slicing. (**c**) Optical microscopy image of measured device.

Figure 3 shows I_DS_–V_DS_ and I_DS_–V_GS_ curves of a HEMT device with a source–drain distance of 9 µm, a gate width of 100 µm, and a source–gate distance of 2 µm in the on-wafer state and after thinning by laser slicing. It can still be operated as a HEMT device even after laser slicing. There were insignificant changes in electrical characteristics before and after slicing, with only a slight decrease in I_DS_ in the HEMT device after slicing. As shown in Fig. 3b, the on-resistance appears to increase from 12.9 Ωmm (on-wafer) to 13.8 Ωmm (as-sliced) with fully opened gate (V_GS_ = 10 V). As reported in reference^33^, the depth of damage caused by laser slicing is around 40 µm from the sliced surface. The HEMT structures in this study are 50 µm away from the sliced surface; thus, significant damage in the form of cracks or alteration of GaN should not reach the devices. Therefore, we considered that the changes in electrical characteristics might be due to the deformation of the GaN chip caused by thinning.

**Figure 4:**
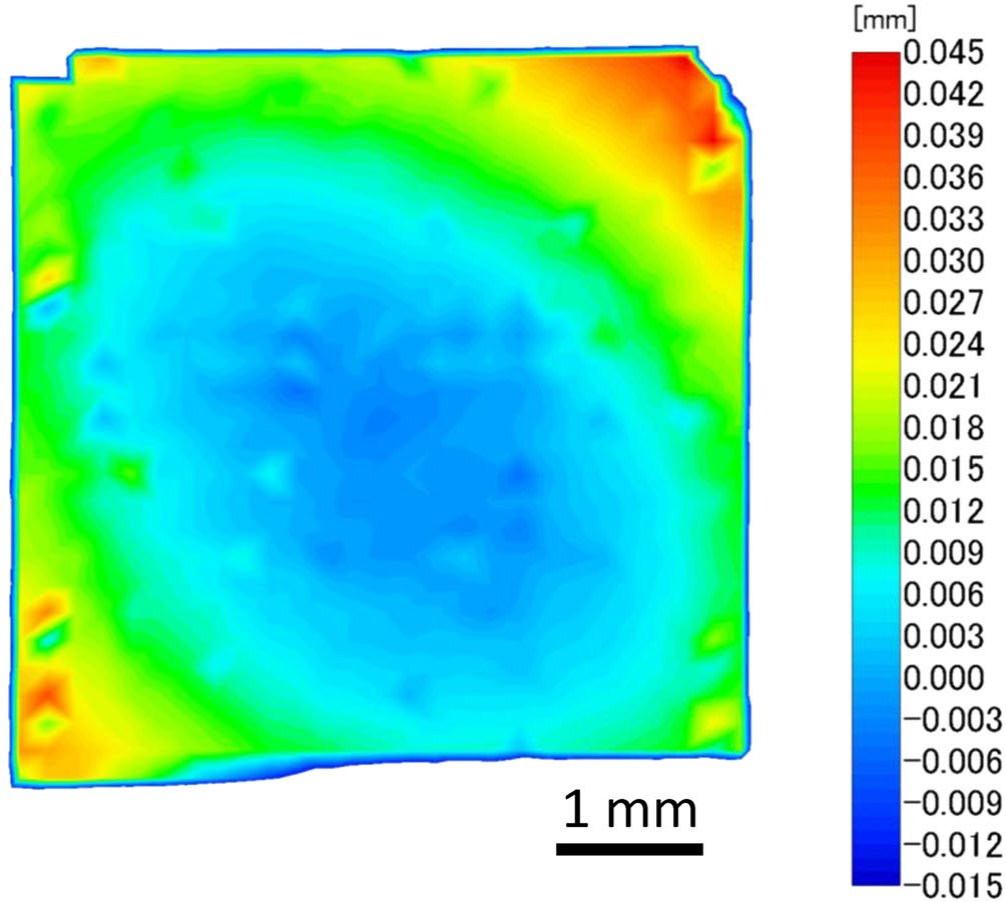
Height distribution of HEMT sample surface.

**Figure 5:**
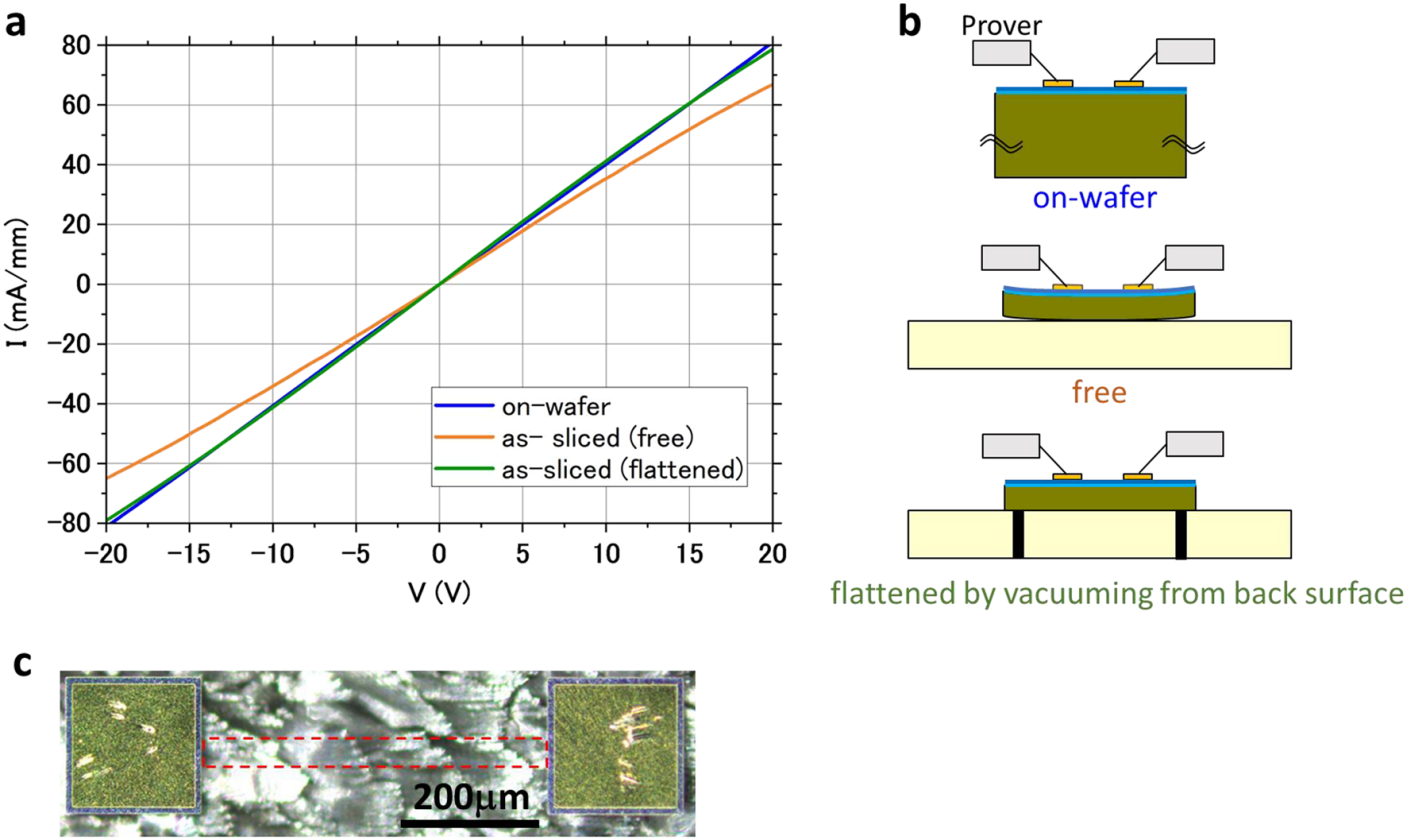
(**a**) I–V characteristics of 2DEG channel of various shapes. (**b**) Schematic of each measurement. (**c**) Optical microscopy image of measured device. The red dashed rectangle indicates the 2DEG channel region.

Figure 4 shows the height distribution of a HEMT sample surface measured using a laser height meter. As shown in Fig. 4, the sample is warped in a downward convex direction. This warpage is considered to be caused by the following factors, which appear as a change in shape caused by the thinning. The compressive stress on the device side of the GaN chip is caused by metal cooling and shrinking after deposition, the film stress of SiN deposited as a protective film, and the AlGaN layer. The tensile stress on the back surface of the GaN chip is caused by the Twyman effect^41,42^ with the rough as-sliced back surface. All of these caused the warping of the chip in a downward convex direction. On the other hand, the only thing that warped the chip in an upward convex direction is the tensile stress on the device side of the GaN chip due to the expansion of the surface side caused by boron ion implantation for electrical isolation. It is difficult to quantitively compare the effects of these factors, but the results show that the compressive stress on the surface side seems to be large and the warp is in a downward convex direction. As discussed in references^43–46^, the warping occurs in the direction in which the strain relaxes at the AlGaN/GaN interface and the electron density in two-dimensional electron gas (2DEG) decreases, which results in an increase in the resistance of the HEMT device and a decrease in I_DS_. To determine whether chip warpage affect the resistance, we measured the I–V characteristics of a 50-µm-wide, 500-µm-long 2DEG channel of various shapes, which was also on a laser-sliced chip. The resistance component of this device is mostly accounted for by 2DEG channel resistance; therefore, the resistance of this device is more sensitive to a change in 2DEG channel resistance than to the change in the resistance of the measured HEMT device. Figure 5 shows the I–V characteristics for each shape. As shown in Fig. 5, the resistance increased after laser slicing from 247 Ωmm (on-wafer) to 304 Ωmm; however, the resistance returned to almost the same value (254 Ωmm) as that under the on-wafer condition when the sliced chip was flattened. This means that the changes in electrical characteristics before and after laser slicing were almost entirely due to the warping of the sample and no significant irreversible changes in electrostatic properties occurred. The change in shape can be corrected by attaching the chip to a heat spreader with sufficient composition.

## Conclusion

We demonstrated the thinning of GaN-on-GaN HEMTs to 50 µm thickness by laser slicing. We also confirmed that GaN-on-GaN HEMTs remained operational even after laser slicing and there were no significant changes in the electrostatic properties of the HEMTs after slicing, except for the warping induced by the thinning. This means that the laser slicing process can be applied even after device fabrication. It can also be used as a semiconductor process for fabricating thin devices with thicknesses on the order of 10 µm without polishing the GaN substrate.
