# Author Correction: Laser slice thinning of GaN-on-GaN high electron mobility transistors

**DOI:** 10.1038/s41598-022-12628-0

**Published:** 2022-05-17

**Authors:** Atsushi Tanaka, Ryuji Sugiura, Daisuke Kawaguchi, Yotaro Wani, Hirotaka Watanabe, Hadi Sena, Yuto Ando, Yoshio Honda, Yasunori Igasaki, Akio Wakejima, Yuji Ando, Hiroshi Amano

**Affiliations:** 1 Center for Integrated Research of Future Electronics (CIRFE), Institute of Materials and Systems for Sustainability (IMaSS), Nagoya University, Aichi, 464-8601 Japan; 2 National Institute for Materials Science, Tsukuba, 987-6543 Japan; 3 Research & Development Department, Electron Tube Division, Hamamatsu Photonics K. K., Shizuoka, 438-0193 Japan; 4 Department of Electrical and Mechanical Engineering, Nagoya Institute of Technology, Aichi, 466-8555 Japan

Correction to: *Scientific Reports* 10.1038/s41598-022-10610-4, published online 05 May 2022.

The original version of this Article contained a repeated error where the symbol for micrometres “µm” was incorrectly given as millimetres “mm” in the Introduction, Experiments, Results and discussion, Figure 2 legend and the Conclusion. This error has been corrected throughout the text.

The original Article has been corrected.

